# Dr. Edwin M. Stillman

**Published:** 1899-01

**Authors:** 


Dr. Edwin M. Stillman died at his home in Andover, N. Y., December 5, 1898. He was 57 years old. He was born in Almond, and always lived in Allegany county. Dr. Stillman was graduated from the Buffalo Medical College in 1865 and practised six months in Almond, four years in Andover, six years in Alfred and since 1874 in Andover. He was a member of the Medical Society of the County of Allegany and served as postmaster of Andover for three years. He leaves a widow and a daughter, Mrs. Richardson of Andover.

